# Differential Adipose Tissue Gene Expression Profiles in Abacavir Treated Patients That May Contribute to the Understanding of Cardiovascular Risk: A Microarray Study

**DOI:** 10.1371/journal.pone.0117164

**Published:** 2015-01-24

**Authors:** Mohsen Shahmanesh, Kenneth Phillips, Meg Boothby, Jeremy W. Tomlinson

**Affiliations:** 1 School of Clinical and Experimental Medicine, College of Medical and Dental Sciences, University of Birmingham, Birmingham, United Kingdom; 2 ILS Genomics (formerly Beckman Coulter Genomics), Morrisville, North Carolina, United States of America; 3 University Hospital Birmingham NHS Foundation Trust, HIV Medicine, Birmingham, United Kingdom; 4 Oxford Centre for Diabetes Endocrinology and Metabolism, University of Oxford, Headington, Oxford, United Kingdom; University of Cape Town, SOUTH AFRICA

## Abstract

**Objective:**

To compare changes in gene expression by microarray from subcutaneous adipose tissue from HIV treatment naïve patients treated with efavirenz based regimens containing abacavir (ABC), tenofovir (TDF) or zidovidine (AZT).

**Design:**

Subcutaneous fat biopsies were obtained before, at 6- and 18–24-months after treatment, and from HIV negative controls. Groups were age, ethnicity, weight, biochemical profile, and pre-treatment CD4 count matched. Microarray data was generated using the Agilent Whole Human Genome Microarray. Identification of differentially expressed genes and genomic response pathways was performed using limma and gene set enrichment analysis.

**Results:**

There were significant divergences between ABC and the other two groups 6 months after treatment in genes controlling cell adhesion and environmental information processing, with some convergence at 18–24 months. Compared to controls the ABC group, but not AZT or TDF showed enrichment of genes controlling adherence junction, at 6 months and 18–24 months (adjusted p<0.05) and focal adhesions and tight junction at 6 months (p<0.5). Genes controlling leukocyte transendothelial migration (p<0.05) and ECM-receptor interactions (p = 0.04) were over-expressed in ABC compared to TDF and AZT at 6 months but not at 18–24 months. Enrichment of pathways and individual genes controlling cell adhesion and environmental information processing were specifically dysregulated in the ABC group in comparison with other treatments. There was little difference between AZT and TDF.

**Conclusion:**

After initiating treatment, there is divergence in the expression of genes controlling cell adhesion and environmental information processing between ABC and both TDF and AZT in subcutaneous adipose tissue. If similar changes are also taking place in other tissues including the coronary vasculature they may contribute to the increased risk of cardiovascular events reported in patients recently started on abacavir-containing regimens.

## Introduction

Antiviral therapies for HIV infection have been associated with abnormalities in serum lipids,[1] an increased incidence of type 2 diabetes mellitus,[2] and increased cardiovascular disease.[3–7] However the metabolic and adipose tissue effects of antiretroviral regimens are not the same with all regimens.[8] Regimens containing tenofovir disoproxil fumarate (TDF) have demonstrated less effect on serum lipids and subcutaneous adipose tissue than those containing zidovidine (AZT).[9] Moreover treatment with abacavir-containing regimens has been associated with increased risk of cardiovascular disease in some studies [10–14] but not others [15–18]

We have previously reported changes in the expression of genes controlling lipid metabolism and mitochondrial respiratory chain in biopsies taken from patients 6 and 18–24 months after starting antiretroviral regimens containing different nucleoside analogue regimens combined with efavirenz. [19,20] Here we report results from a microarray study performed on subcutaneous adipose tissue biopsies from the same study.

## Patients and Methods

**Figure 1:**
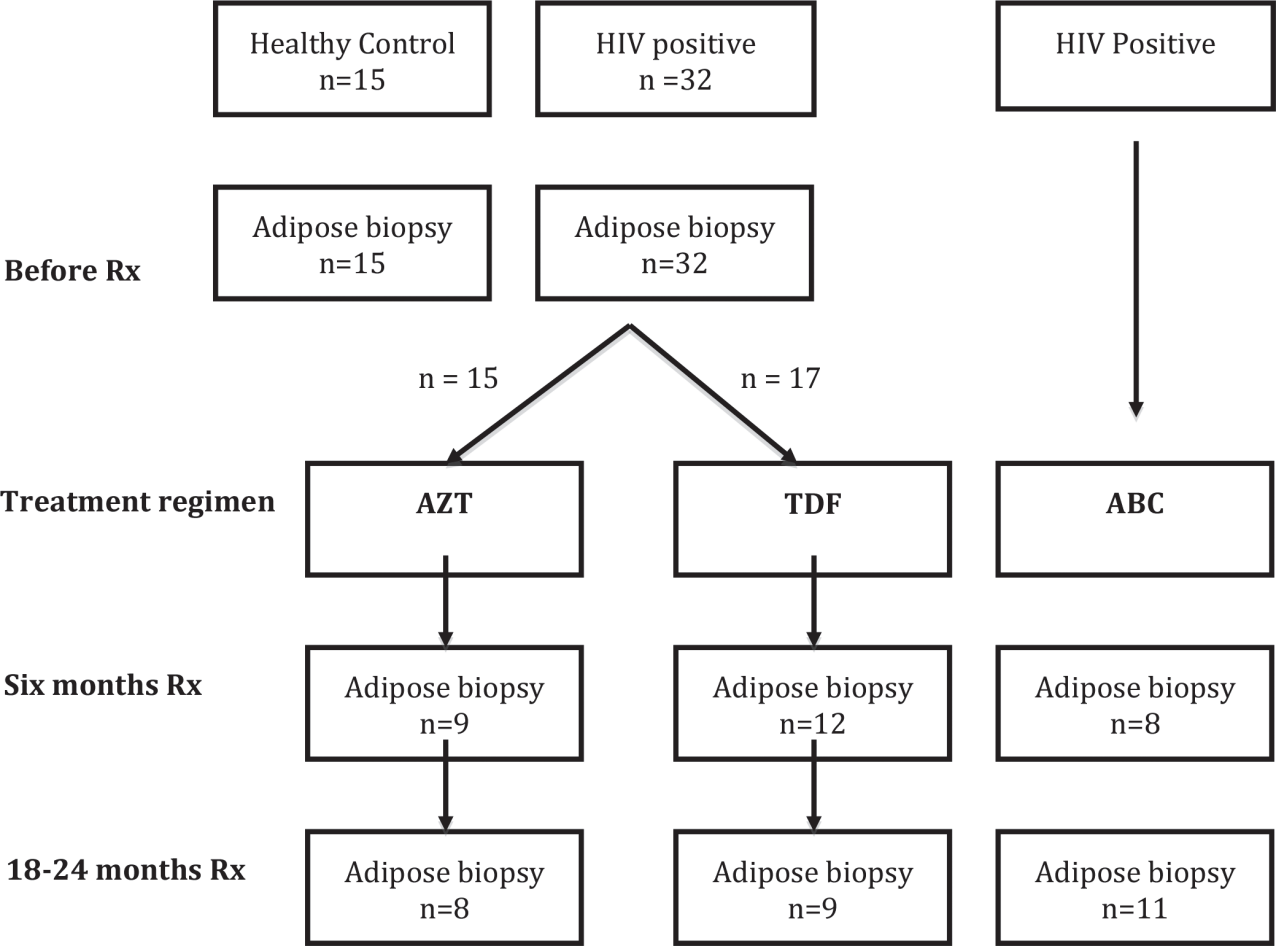
Flow chart of study design. Rx = treatment; AZT = zidovdine and lamivudine; TDF = tenofovir and emtricitabine; ABC = abacavir and lamivudine. All three groups were also taking efavirenz; n = number of samples available for microarray at each time point.

Details of the patients have been previously reported and the design of the study is summarised in Fig. 1. [19,20] Briefly, the study was undertaken in two parts. In the first part iliac crest fat biopsies were performed on 32 HIV-positive patients naïve to antiretroviral therapy who were randomised to receive either zidovidine (AZT)/lamivudine (3TC n = 15) in fixed dose preparation or tenofovir (TDF)/emitricitabine (n = 17), again in fixed dose preparation. Both groups also took efavirenz (EFV). All patients underwent extensive biochemical screening, tests of body fat distribution (whole body DEXA and abdominal CT scans) and iliac crest biopsy under local anaesthesia, as previously described. [19] Tests were performed before starting treatment, at six months and again at 18–24 months after initiating therapy. 15 HIV negative controls underwent similar investigations.

In the second non-randomised part of the study patients who were on their initial antiretroviral regimen containing abacavir (ABC) and 3TC in fixed-drug combination plus EFV for 6 months (n = 8) or 18–24 months (n = 11) were also tested in a similar way. Patients and controls from both parts of the study were matched for age, ethnicity, gender, weight, and BMI. None had ever had an AIDS defining disease. All three HIV groups were also matched with regards to pre-treatment CD4 count, viral load [19,20] and family history of diabetes and cardiovascular disease. The median age (range) for the controls was 37 (22–55), for AZT 33 (22–62), for TDF 35.5 (23–53) and for ABC 37 (21–62). The median (IRQ) CD4 count before treatment were AZT 187 (153–268), TDF 234 (163–253) and ABC 215 (75–306). Pre-treatment viral loads log_10_ (IRQ) were 5.1 (4.4–5.7), 4.6 (4.2–5.3) and 4.5 (4.4–4.9) respectively for AZT, TDF and ABC. All but two patients (both on AZT) had undetectable viral load when tested at 6 month following treatment (209 and 109,638 copies/ml) but all had undetectable viral loads at 18–24 m. Both admitted to poor compliance. None developed any clinical evidence of lipodystrophy during the period of study and post treatment weight, BMI, DEXA and abdominal CT results were not significantly different between groups at both time points after treatment. Serum lipids, fasting glucose and other parameters were similar between groups at each time point.[19,20]

Samples for microarray were available from 103 biopsy specimens (Fig. 1). There was no significant difference in weight, BMI, central and peripheral fat, and visceral and subcutaneous fat, or blood biochemistry in the patients undergoing microarray study at any time point.

### Microarray methods

RNA was extracted for microarray and array data generated using a Qiagen RNeasy Lipid Tissue Mini Kit. Total RNA was converted into labeled cDNA using the WT-Ovation Pico RNA Amplification System and samples were hybridized to an Agilent Whole Human Genome Microarray 4×44K (AMADID 014850, Agilent Technologies). The Agilent arrays were scanned with the Agilent Technologies DNA Microarray Scanner G2505C with default settings. Scanned images were extracted and initial quality control performed in Agilent Feature Extraction (AFE) software version 10.7.3.1. Additional quality assessment was performed using the R *arrayQualityMetrics* package.[21] As no major experimental problems could be detected, all microarrays were included in subsequent analysis steps. The dataset was read into R and processed using the *Agi4x44PreProcess* package with options to use the AFE *Processed Signal* as foreground signal and *BG Used* signal as background adjusted signal.[22] The data were then normalized by the *quantile* method using the *limma* function normalize Between Arrays.[23] Probeset filtering was performed using the *Agi4x44PreProcess* filter method using AFE provided flags to identify features with quantification errors of the signal, reducing the 45,015 features on the Agilent Whole Human Genome Microarray 4×44K array by 34%, or 15,339 features. Residual technical batch effects were corrected using the ComBat method implemented in the *SVA* R package.[24]

Since patients were not randomised to the ABC treatment all samples were treated as unrelated. Gene expression differences between treatment arms were assessed using the linear modeling features implemented in the limma package in R[25]. Multiple testing across contrasts was performed using the limma global method with Benjamini-Hochberg approach for control of false discovery rate (FDR). Gene set enrichment analysis of GO terms and KEGG pathways was performed using the conditional hypergeometric test implemented in the GOstats R package[26]. Statistical significance of gene set enrichment was performed using the Benjamini-Hochberg correction method implemented in the EMA R package [27]. Microarray data have been submitted to the Gene Expression Omnibus (GEO) and are available under NCBI accession number GSE 62117

### Ethics Statement

The study was approved by the South Birmingham Research Ethics Committee (Reference 05/Q2707/40) and the Medicines and Healthcare Products Regulatory Agency—MHRA (Eudract number 2005/004021-26). All patients signed an informed consent to participate in the study.

## Results

### Global Gene Expression

**Figure 2:**
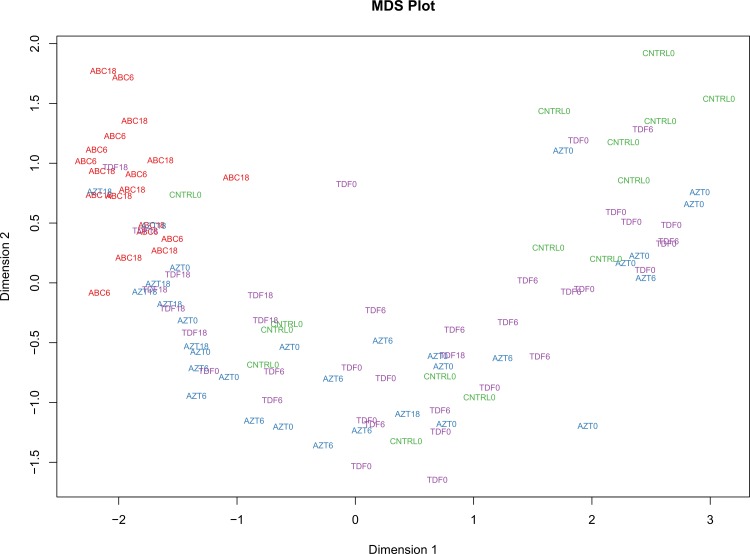
The multi-dimensional scaling plot (MDS) shows that by and large ABC 6 and ABC 18–24 samples are separated from other samples in the study. Distances in the plot represent root-mean-square deviation (Euclidean distance) for the top 500 genes that best distinguish those samples.

Multi-dimensional scaling (MDS) on all 103 samples was performed using profile level gene expression data from individual samples to see if subjects group according to treatment, time points or both. Distances were plotted using the limma plotMDS function with default parameters. Along the first dimension there was a clear separation of samples by samples time whereas in the second dimension the greatest distance was between the ABC treatment groups (6 m and 18–24 m) versus the other treatment groups including the untreated patients and the controls (Fig. 2).

Groups of differentially expressed genes were next generated using the R/Bioconductor limma library. Statistical criteria for identification of differentially expressed genes were FDR adjusted P* < 0.01 and fold change greater than 2.0. The cross sectional comparisons Table 1 showed the difference between groups was particularly striking at 6 months with ABC showing 4,185 differentially expressed genes (DEGs) compared to controls while the number of DETs for AZT and TDF were 895 and 381 respectively. Gene expression between the three groups at 18–24 months appear to have converged. At 18–24 m there were only 5 DETs between AZT and TDF, 88 between AZT and ABC and 251 between TDF and ABC (Table 1).

**Table 1:** Differentially expressed sequences between groups at 6 months and 18–24 months.

	**AZT 6**	**TDF6**	**ABC 6**	**AZT 18–24**	**TDF 18–24**	**ABC 18–24**
**Control**	895	381	4146	3255	3322	4185
**AZT 6**		90	3434			
**TDF 6**			4288			
**AZT 18–24**					5	88
**TDF 18–24**						251

### Gene enrichment analysis

#### a. Comparison with controls

To gain mechanistic insight statistical list DEGs for each treatment contrast were tested for association to Gene Ontology (GO) terms and KEGG terms using the conditional hypergeometric test algorithm implemented in R package *GOStat*s (Falcon and Gentleman, 2007). Statistical significance of gene set enrichment was performed using the Benjamini-Hochberg correction method. Selected KEGG pathways showing over-representation in the three treatment groups compared to the controls are shown in the (Table 2).

**Table 2:** Selected KEGG gene pathways relating to cell communication and environmental information processing that were significantly enriched compared to HIV negative controls.

	**Time**	**Term**	**KID**	**Adjusted P**	**OR**	**Expected count**	**Observed count**
**Control—ABC**	6 m	ECM-receptor interaction	4512	0.036	1.80	16	25
		Focal adhesion	4510	0.036	1.40	41	53
		Tight junctions	4530	0.043	1.50	24	32
		Adherence Junction	4520	0.043	1.62	16	23
**Control—ABC**	18–24 m	Wnt signalling pathway	4310	0.045	1.49	27.1	36
		Adherence Junction	4520	0.045	1.65	16.2	23
		ECM-receptor interaction	4512	0.046	1.65	16.2	23
**Control—AZT**	6m	Cytokine-cytokine receptor interaction	4060	0.03	3.02	2.17	6
**Control – AZT**	18–24 m	Neuroactive ligand-receptor interaction	4080	<0.0001	2.78	26.1	54
		Cytokine-cytokine receptor interaction	4060	0.019	1.61	25	36
		ECM-receptor interaction	4512	0.034	1.78	11.5	18
**Control—TDF**	6 m	Cytokine-cytokine receptor interaction	4060	0.038	6.90	0.58	3
**Control—TDF**	18–24 m	Neuroactive ligand-receptor interaction	4080	<0.0001	2.67	25	51
		Cytokine-cytokine receptor interaction	4060	0.01	1.76	24	37
		ECM-receptor interaction	4512	0.021	2.03	11.1	19
		VEGF signalling pathway	4370	0.029	1.88	10.4	17
		WNT signalling pathway	4310	0.04	1.54	18.5	26

KID = Kegg identity; OR = odds ratio; Time = time after treatment

Adherence junction was significantly enriched compared to controls in the ABC group at both time points but not in the other two treatment groups. Six months after treatment 24 genes were up-regulated and 8 genes were down regulated compared to controls in the ABC group (<0.01). At 18–24 months after treatment the corresponding figures were 22 up-regulated and 7 down regulated (p<0.01) Individual adherence junction genes significantly up-regulated or down-regulated in the ABC treatment arm compared to controls are tabulated in Table 3.

**Table 3:** Expression of genes controlling adherence junction in ABC treated group in comparison with controls at 6 months and 18–24 months after treatment.

**Time on treatment**	**Upregulated**	**Down regulated**	**Adjusted p value**
6 months	ACTN1, CSNK2A1, CTNNA1, CTNNB1, CTNND1, EGFR, EP300, FGFR1, INSR, IQGAP1, NLK, PTPN1, PTPRB, PTPRF, PTPRJ, PTPRM, RHOA, SMAD2, SORBS1,TCF7L1, TCF7L2, TGFBR1, TJP1,VCL	CLDN3, CLDN4, CLDN5, MAP3K3, PVRL1, RAC2, SNAI1, SNAI2,	– <0.01–<0.0001
18–24 months	ACTN1, CSNK2A1, CTNNA1, CTNNB1, EGFR, FGFR1, INSR, IQGAP1, MAP3K3, NKL, PTPN1, PTPRB, PTPRF, PTPRJ, PTPRM, RHOA, SMAD2, SORBS1, TCF7L2, TGFBR1, TJP1,VCL	CLDN3, CLDN4, CLDN5, MAP3K3, RAC2, SNAI1, SNAI2	<0.01–<0.0001

ACTN1 = actinin, alpha 1; CSNK = casein kinase; CLDN = claudin, CTNN = catenin (cadherin-associated protein); EGFR = epidermal growth factor receptor, EP300 = E1A binding protein p300; FGRF1 = fibroblast growth factor receptor 1; INSR = insulin receptor; IQGAP 1 = IQ motif containing GTPase activating protein 1; MAP3K7 = mitogen-activated protein kinase kinase kinase 7; MLLT4 = myeloid/lymphoid or mixed-lineage leukemia (trithorax homolog, Drosophila); translocated to, 4; NLK = nemo-like kinase; PTPRB, PTPRF, PTPRFJ, or PTPRFM = protein tyrosine phosphatase, receptor B, F, J, or M; PVRL 1 = poliovirus receptor-related 1 (herpesvirus mediator C); RAC2 = ras-related C3 botulinum toxin substrate; RHOA = ras homolog family member A; SMAD = SMAD family member; SNAI1 and SNAI2 = snail family zinc finger; SORBS1 = sorbin and SH3 domain containing 1 and 2; TCF7L1 and TCF7L2 = transcription factor 7-like; TGFBR = transforming growth factor 1 and 2, beta receptor; VCL = vinculin.

Focal adhesions and tight junctions were also significantly enriched compared to the controls in the ABC treated group at 6 months but not in the other treatment groups. (Table 2)

GO terms significantly enriched with ABC but not the other treatment groups at 6 months after treatment include cell adhesion (GO:7155, OR 1.3, p = 0.04), apical junction assembly (GO:43297, OR 2.8, p = 0.03), cell-cell junction assembly (GO:7043, OR 1.8, p = 0.04), cell-cell junction organisation (GO:45216, OR 1.4, p = 0.04), and cell matrix adhesion (GO:7160, OR1.7, p = 0.04). At 18–24 months Go terms significantly enriched with ABC treatment alone include cell-cell—junction assembly (OR 1.4, p = 0.04), cell-cell signaling (GO:726, OR 1.8, p<0.002) and for ABC and TDF signaling (GO:23052, OR1.2, p = 0.003).

#### b. Comparison between treatment arms at each time point

**Figure 3:**
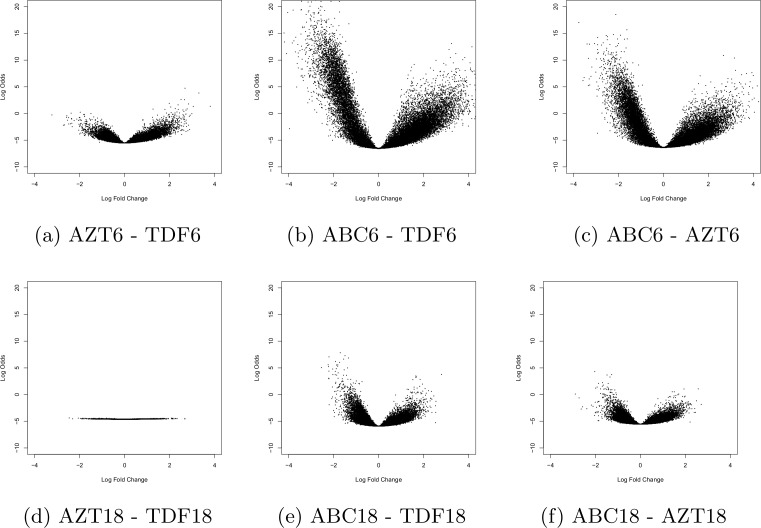
Volcano plot showing significance versus fold changes in the y- and x-axis respectively for the three treatment groups at 6 months on top row (a-c) and 18 months on bottom row (d-f). For each comparison the first term is the numerator and the second denominator.

We next performed enrichment analysis using statistical list of DEG from contrast of the three different treatment regimens at 6 and 18–24 months (excluding the HIV negative samples) in a cross sectional analysis. A Venn analysis was performed for each time point and Volcano plots were created filtering by fold change >1.5 and Benjamini-Hochberg corrected p-value <0.05 at 6 months and 18–24 months (Fig. 3).

Six months after treatment enrichment of gene pathways involved with WNT signalling and leukocyte transendothelial migration were seen in ABC compared to patients receiving TDF and neuroactive ligand receptor interaction, leukocyte transendothelial migration and cytokine-cytokine receptor interactions compared to patients receiving AZT (Table 4).

**Table 4:** Selected KEGG gene pathways overrepresented in a cross sectional comparison of ABC versus AZT and TDF.

	**Time**	**Pathway**	**KID**	**Adj p value**	**OR**	**Expected count**	**Observed count**
**ABC-TDF**	6 m	Leukocyte transendothelial migration	4670	0.03	1.6	22.3	32
		WNT signalling pathway	4301	0.01	1.8	27.2	37
		ECM-receptor interaction	4512	0.04	1.6	16.3	23
**ABC-AZT**	6m	Neuroactive ligand receptor interaction	4080	<0.0001	2.6	20.1	48
		ECM-receptor interaction	4512	0.02	1.9	12.3	20
		Leukocyte transendothelial migration	4670	0.04	1.7	12.1	19

KID = KEGG pathway ID; OR = odds ratio; Time = months on treatment; adj = adjusted

KEGG pathways significantly over expressed when ABC was compared with the combined other two treatment groups at six months (intersection in Venn analysis) included ECM receptor interaction, leukocyte transendothelial migration, tight junction, focal adhesion, WNT signalling, and cell adhesions (p<0.02).

Go terms over represented in ABC compared to AZT treatments at 6 months include signalling (GO:23052, OR1.3, p = 0.0003), cell-cell signalling (GO:726, OR 1.7, p = 0.003). Most of the above pathways are involved with cell processes and cell communication (adherence junction, tight junction, gap junction, focal adhesion -or environmental information processing (WNT signalling, ECM receptor interaction, neuroactive ligand interactions, cell adhesion molecules, leukocyte transendothelial migration).

The differences in the expression of genes relating to tight junctions and gap junction were particularly apparent between ABC and TDF at 6 m. Compared to TDF, 27 ABC genes controlling tight junctions were significantly up-regulated and 10 down-regulated (p<0.01). For gap junction genes the corresponding numbers were 19 up-regulated and 8 down regulated (p = 0.01). Similarly at 6 months compared to TDF and AZT 12 and 13 ABC genes respectively controlling AJ were up-regulated and 8 and 6 genes down-regulated (p<0.01) while at 18–24 months only one gene in each treatment groups was significantly up-regulated and none down-regulated.

At 18–24 months there were insufficient genes for a KEGG pathways or GO term gene enrichment analysis.

#### c. Comparison with HIV treatment naïve

Enrichment analysis was next performed using statistical list of DEG in the three treatment groups and HIV infected treatment naïve subjects. The AZT and TDF pre-treatment samples were combined (n = 31) to form the HIV treatment naive group. These were then contrasted with the three treatment groups at 6 m and 18–26 months using limma R/Bioconductor limma library. A Venn analysis was performed for each time point and the unique gene sets expressed were tested for over representation analysis of KEGG pathways and GO-terms using the standard Hypergeometric test.

Significant number of genes were only available for a comparions between treatment groups six months after treatment, but not at the later time point. There was enrichment of the MAPK signalling pathway for ABC group compared to HIV treatment naïve patients (p<0.05) which was not seen with the other two groups. None of the pathways relating to environmental processing or signal transduction was enriched in the TDF and AZT treatment groups.

#### d. Individual gene analysis

In view of the findings showing enrichment of pathways relating to endothelial function we examined a number of genes relating to endothelial adhesion molecules some of which that had previously been reported to be affected by HIV and its treatment as well as some genes involved with tight junction, adherence junction, leukocyte transendothelial migration, and cytokine-cytokine receptor interaction. As can be seen (Table 5) there was over expression of some genes with ABC treatment in comparison with AZT and TDF. Differences between the three groups disappeared in all but one of the genes examined at 18–24 months.

**Table 5:** Expression of selected genes involved with endothelial cell adhesion molecules, tight junction and adherence junction.

**Gene**	**ABC 6m vs cont**	**ABC 18–24m vs cont**	**ABC 6m vs AZT 6m**	**ABC 6m vs TDF 6m**	**ABC 18–24m vs AZT 18–24m**	**ABC 18m vs TDF 18–24m**	**Pathway**
**ICAM 1**	1.5 p = 0.008	1.7 p = 0.001	NS	NS	NS	NS	CAM
**ICAM 2**	NS	NS	NS	1.9 p = 0.01	NS	NS	CAM
**ICAM 4**	−1.9 p<0.0001	−1.9 p<0.0001	−1.0 p = 0.5	−1.3 p = 0.005	NS	NS	CAM
**ICAM5**	−3.2 p<0.0001	−2.6 p<0.0001	−2.2 p<0.0001	−2.8 p<0.0001	NS	NS	CAM
**TICAM1**	−3.0 p<0.0001	−2.2 P<0.0001	−1.7 p = 0.002	−2.0 p<0.0001	NS	NS	TLR, NFkB
**TICAM2**	3.0 p<0.0001	2.8 p<0.0001	NS	2.0 p<0.0001	NS	NS	TLR, NFkB
**SELPLG**	−2.6 p<0.0001	−2.0 p<0.0001	−1.7 p = 0.001	−2.2 p<0.0001	NS	NS	CAM
**CD40**	−2.2 p<0.0001	−1.9 p<0.0001	−2.1 p<0.0001	2.7 p<0.0001	NS	NS	CAM, CCR, NFkB
**JAM-2**	2.9 p = 0.0005	2.6 p = 0006	NS	1.8 p = 0.005	NS	NS	TJ, LTM
**ITGB-1**	2.2 P<0.0001	1.9 p<0.0001	1.6 p = 0.01	2.0 p = 0.002	NS	NS	LTM
**VAPA**	1.1 p = 0.001	1.2 p = 0.001	1.2 p = 0.002	1.2 p = 0.0005	NS	NS	TJ
**PPP2R4**	−1.7* p<0.0001	−1.4** p = 0.0002	−1.7 p = 0.001	−1.9 p<0.0001	NS	NS	TJ
**CLDN3**	−2.0 p<0.0001	−2.2 p<0.0001	−1.5 p = 0.001	−2.0 p<0.0001	NS	NS	CAM, AJ, TJ
**CLDN 4**	−1.9 p<0.0001	−1.6 p<0.0001	−2.0 p = 0.003	−2.2 p<0.0001	NS	NS	CAM, TJ, LTM
**CLDN5**	−2.2 p<0.0001	−1.5 p<0.0001	−1.7 p<0.0001	−2.0 p<0.0001	NS	NS	CAM, AJ, TJ, LTM
**CTNNA1**	1.8 p = 0.001	1.8 p = 0.0004	1.6 p = 0.01	1.9 p = 0.001	NS	NS	AJ, TJ, AJ, LTM
**CTNNB1**	1.5 p = 0.03	1.8 p = 0.002	1.8 p = 0.0006	1.6 p = 0.005	1.6 p = 0.005	NS	AJ, TJ, LTM
**ERBB2**	−2.4 p<0.0001	−2.8 p<0.0001	−1.8 p<0.0004	−2.3 p<0.0001	NS	NS	AJ, TJ
**MLLT4**	−2.3 p = 0.002	−2.0 p = 0.0004	−2.4 p = 0.001	−2.8 p = <0.0001	NS	NS	AJ, LTM

Results are shown as fold change of ABC treatment versus other treatment groups. Minus sign denotes down regulation of ABC compared to comparison treatment. P = adjusted significance; ICAM = intracellular adhesion molecule; SELPLG = selectin P ligand; CD40 = TNF receptor superfamily member 5 (CD40); transcript variant 1; JAM = junction adhesion molecule; ITGB1 = integrin, beta 1 (fibronectin receptor, beta polypeptide, antigen CD29 includes MDF2, MSK12);VAPA = Homo sapiens VAMP (vesicle-associated membrane protein)-associated protein A. PPP2R4 = Homo sapiens protein phosphatase 2 (formerly 2A), regulatory subunit 4; CLDN = claudin mRNA; CTNNA1and B1 Catenin (cadherin-associated protein) alpha1and beta 1; ERBB2 = erythrobalstic leukaemia viral oncogene homolog2, neuro/glioblastoma derived oncogene homolog (avian), transcript variant 2, mRNA; MLLT4 = myeloid leukemia/lymphoid or mixed linage leukaemia; translocated to, 4, transcript variant 3, mRNA

* 14 other variants of PPP2A were also significantly upregulated with ABC 6 months compared to the combined AZT and TDF group (p = 0.009–6.8E–10)

** 4 other variants of PPP2A were upregulated (p = 7.0E–10 to p = 0.003) and 6 variants down regulated (p = 0.0001–0.003)

Pathways: AJ = adherence junction; CAM = cell adhesion molecule; CCR = cytokine-cytokine receptor interaction; NFkB = NF kappa B; LTM = leukocyte transendothelial migration; TJ = tight junction; TLR = toll-like receptors

Moreover, when we performed enrichment analysis against the GO term ‘biological adhesions’ (GO:22610) compared to ABC six months after treatment, of the 39 genes expressed, 21 genes in the TDF group and 12 genes in the AZT group were either significantly up-or down regulated (p<0.05). There was no significant difference when AZT and TDF were compared at 6 months. However at 18 m the three regimens had converged such that significant enrichment was only seen in two genes for TDF and one gene for AZT compared to ABC, with the two latter treatment groups again showing no difference when compared against one another. A similar difference was observed when the three treatment groups were compared with controls with 29 genes in GO term ‘biological adhesions’ registered as significant at 6 months for ABC against none for the other two groups while the number of genes being significantly enriched at 18–24 months against the treatment naïve patients were 27, 17 and 15 for ABC, TDF and AZT respectively. A selected list of GO terms relating to cell adhesion comparing controls with the three treatment groups as well as between group comparisons are shown in Table 6. Compared to controls 48 and 17 genes controlling GO terms ‘cell junction assembly’ and ‘apical junction’ respectively were up-regulated and 17 and 4 genes were down-regulated (p<0.01). Similarly, for GO term ‘cell adhesion’, 172 genes were up-regulated and 17 were down-regulated in ABC-treated patients for 6 months compared to controls.

**Table 6:** Selected GO terms relating to cell adhesion.

**Go term**	**Go ref**	**Cont vs ABC 6m**	**Cont vs AZT 6m**	**ABC vs AZT 6m**	**ABC vs TDF 6m**	**Cont vs ABC 18–24m**	**Cont vs AZT 18–24m**	**Cont vs TDF 18–24m**
Biological adhesion	22610	OR 1.3 p = 0.03		OR 1.3 p = 0.01			OR 1.3 p = 0.009	OR 1.5 p = 0.0008
Cell adhesion	7155	OR 1.3 P = 0.03		OR 1.3 p = 0.01		OR 1.2 p = 0.07		OR 1.5 p = 0.0009
Cell-cell adhesion	16337	OR 1.4 p = 0.04	OR 2.3 p = 0.04	OR 1.4 p = 0.03			OR 1.5 p = 0.01	OR 1.5 p = 0.02
Regulation of cell-cell adhesion	22407	OR 1.9 p = 0.04	OR 7.2 p = 0.04	OR 1.7 p = 0.02		OR 1.8 p = 0.04	OR 2.0 p = 0.026	OR 1.9 p = 0.035
Apical junction assembly	43297	OR 2.8 p = 0.02			OR 2.0 p<0.04			
Cell-cell junction assembly	7043	OR 1.8 p = 0.4			OR 1.8 p = 0.04	OR 1.4 p = 0.04		
Cell-cell junction organisation	45216	OR 1.4 p = 0.04						

None of the GO terms appeared for control vs TDF at 6 months. Cont = control; GO ref = GO term reference; m = months on treatment; OR = odds ratio; p = adjusted p value

A complete list of Kegg pathways that are enriched for all three treatment groups at different times after initiating treatment compared to controls is shown on Table 7.

**Table 7:** KEGG gene pathways significantly enriched compared to HIV negative controls.

**Treatment**	**Time**	**KID**	**Term**	**Expected Count**	**Cnt**	**OR**	**P value**	**adjusted P value**
Control—ABC	6 m	604	Glycosphingolipid biosynthesis—ganglio series	2.89	8	6.35	0.0021	0.0189
		5412	Arrhythmogenic right ventricular cardiomyopathy (ARVC)	13.48	23	2.23	0.0034	0.0189
		4512	ECM-receptor interaction	16.37	25	1.86	0.0124	0.0363
		510	N-Glycan biosynthesis	11.07	18	2.05	0.0161	0.0363
		600	Sphingolipid metabolism	7.46	13	2.29	0.0210	0.0363
		4510	Focal adhesion	41.15	53	1.44	0.0212	0.0363
		4622	RIG-I-like receptor signaling pathway	11.55	18	1.91	0.0255	0.0363
		3450	Non-homologous end-joining	2.65	6	3.80	0.0285	0.0363
		4360	Axon guidance	24.31	33	1.55	0.0297	0.0363
		4530	Tight junction	24.07	32	1.50	0.0423	0.0433
		4520	Adherens junction	16.37	23	1.63	0.0433	0.0433
								
Control—ABC	18–24 m	4360	Axon guidance	24.05	40	2.14	0.0003	0.0034
		510	N-Glycan biosynthesis	10.95	19	2.28	0.0062	0.0404
		4141	Protein processing in endoplasmic reticulum	35.95	48	1.52	0.0142	0.0457
		600	Sphingolipid metabolism	7.38	13	2.33	0.0192	0.0457
		62	Fatty acid elongation in mitochondria	1.90	5	5.35	0.0221	0.0457
		3450	Non-homologous end-joining	2.62	6	3.86	0.0271	0.0457
		4310	Wnt signaling pathway	27.14	36	1.49	0.0341	0.0457
		2010	ABC transporters	7.14	12	2.15	0.0354	0.0457
		5200	Pathways in cancer	63.34	76	1.30	0.0371	0.0457
		4520	Adherens junction	16.19	23	1.65	0.0387	0.0457
		4512	ECM-receptor interaction	16.19	23	1.65	0.0387	0.0457
		604	Glycosphingolipid biosynthesis—ganglio series	2.86	6	3.21	0.0435	0.0462
		4610	Complement and coagulation cascades	11.43	17	1.77	0.0462	0.0462
								
Control—AZT	6m	5332	Graft-versus-host disease	0.43	3	8.08	0.0084	0.0300
		4020	Calcium signaling pathway	1.98	6	3.34	0.0136	0.0300
		4970	Salivary secretion	0.97	4	4.55	0.0155	0.0300
		4060	Cytokine-cytokine receptor interaction	2.17	6	3.03	0.0206	0.0300
		5014	Amyotrophic lateral sclerosis (ALS)	0.62	3	5.37	0.0231	0.0300
		4210	Apoptosis	1.13	4	3.86	0.0258	0.0300
		512	Mucin type O-Glycan biosynthesis	0.31	2	7.26	0.0373	0.0373
								
Control—AZT	18–24 m	4080	Neuroactive ligand-receptor interaction	26.07	54	2.78	0.0000	0.0000
		4950	Maturity onset diabetes of the young	3.05	10	6.20	0.0002	0.0017
		4020	Calcium signaling pathway	22.86	36	1.83	0.0025	0.0108
		5410	Hypertrophic cardiomyopathy (HCM)	10.67	20	2.32	0.0027	0.0108
		4975	Fat digestion and absorption	4.91	11	3.03	0.0055	0.0176
		5014	Amyotrophic lateral sclerosis (ALS)	7.11	14	2.48	0.0070	0.0188
		4142	Lysosome	18.29	28	1.75	0.0110	0.0198
		4930	Type II diabetes mellitus	6.77	13	2.39	0.0117	0.0198
		4340	Hedgehog signaling pathway	6.10	12	2.48	0.0122	0.0198
		4060	Cytokine-cytokine receptor interaction	25.06	36	1.61	0.0124	0.0198
		4740	Olfactory transduction	7.11	13	2.22	0.0179	0.0260
		531	Glycosaminoglycan degradation	2.37	6	3.70	0.0204	0.0271
		983	Drug metabolism—other enzymes	4.57	9	2.47	0.0285	0.0347
		4512	ECM-receptor interaction	11.51	18	1.79	0.0304	0.0347
		4974	Protein digestion and absorption	9.31	15	1.86	0.0358	0.0382
		4380	Osteoclast differentiation	18.45	26	1.56	0.0383	0.0383
								
Control—TDF	6m	310	Lysine degradation	0.16	2	14.9	0.0106	0.0388
		4621	NOD-like receptor signaling pathway	0.19	2	11.98	0.0156	0.0388
		4060	Cytokine-cytokine receptor interaction	0.59	3	6.10	0.0192	0.0388
		5131	Shigellosis	0.22	2	10.6	0.0194	0.0388
		53	Ascorbate and aldarate metabolism	0.04	1	33.3	0.0352	0.0417
		4210	Apoptosis	0.31	2	7.46	0.0364	0.0417
		4666	Fc gamma R-mediated phagocytosis	0.33	2	6.99	0.0408	0.0417
		4650	Natural killer cell mediated cytotoxicity	0.33	2	6.90	0.0417	0.0417
								
Control—TDF	18–24 m	4080	Neuroactive ligand-receptor interaction	25.03	51	2.67	0.0000	0.0000
		4610	Complement and coagulation cascades	7.80	20	3.76	0.0000	0.0002
		4020	Calcium signaling pathway	21.94	37	2.00	0.0006	0.0030
		4930	Type II diabetes mellitus	6.50	14	2.81	0.0029	0.0100
		4060	Cytokine-cytokine receptor interaction	24.06	37	1.76	0.0036	0.0100
		4975	Fat digestion and absorption	4.71	11	3.18	0.0040	0.0100
		4512	ECM-receptor interaction	11.05	19	2.03	0.0099	0.0213
		4974	Protein digestion and absorption	8.94	16	2.14	0.0116	0.0218
		4950	Maturity onset diabetes of the young	2.93	7	3.30	0.0179	0.0276
		5217	Basal cell carcinoma	5.69	11	2.38	0.0191	0.0276
		5410	Hypertrophic cardiomyopathy (HCM)	10.24	17	1.93	0.0202	0.0276
		4370	VEGF signaling pathway	10.40	17	1.89	0.0235	0.0294
		4740	Olfactory transduction	6.83	12	2.08	0.0310	0.0351
		604	Glycosphingolipid biosynthesis—ganglio series	1.95	5	3.70	0.0327	0.0351
		4310	Wnt signaling pathway	18.53	26	1.54	0.0407	0.0407

KID = Kegg identity; Cnt = actual count, OR = odds ratio; Time = time after treatment

#### e. Validation of microarray

In order to validate the microarray study we looked at genes that we had previously shown to be significantly up or down-regulated in the adipose tissue by real time PCR on the same subcutaneous fat samples. [19,20] Thus we found that compared to the controls hexose 6-phosphate dehydrogenase (H6PD) was up-regulated (fold change 4.1, p<0.0001) in the AZT treated group as was also found with PCR. Expression of 11-beta hydroxysteroid dehydrogenase-1 (11βHSD-1) was down-regulated compared pre-treatment expression for both AZT and TDF (p = 0.001 and p<0.01 respectively) in keeping with real time PCR. Similarly, uncoupling protein 2 (UCP-2) was up-regulated for AZT treatment group at 18–24 months (p = 0.009) compared to pre-treatment values and UCP-3 down-regulated for both AZT and TDF (p = 0.007 and p = 0.004 respectively), as previously reported for PCR. Cytochrome BB (CYBB) and cytochrome B5 were down-regulated (p = 0.03 and p = 0.009 respectively) after 18–24m AZT treatment. Moreover CYBB was significantly up-regulated in the TDF 18–24m group compared to AZT treatment after 18–24 m (p = 0.001). These findings confirm our previous report that treatment with AZT, compared to TDF appears to impair mitochondrial oxygen transport genes.[19,20]

Biological validation also comes from the observation that KEGG pathway enrichment was observed for fatty acid metabolism (TDF, p = 0.04), glycolysis and gluconeogenesis (TDF, p = 0.04), and steroid hormone biosynthesis (AZT, p = 0.03) at six months. At 18–24 months after treatment PPAR signalling pathway (OR 5.4, p = 0.023) and biosynthesis of fatty acids (OR 9.2, p = 0.026) were enriched in all three treatment groups, an observation in keeping with return to health reported in many studies, and the observed increase in limb and central fat in the first 24 weeks of antiviral therapy.[28] The consistency of the direction of changes in individual genes in the cross-sectional comparisons across the treatment groups at both time intervals (Table 6) strengthens further the validity of the study.

## Discussion

In this study we generated microarray data from mRNA prepared from subcutaneous fat samples from HIV negative controls, treatment naïve HIV infected individuals, and after 6 months and 18–24 months into their initial antiretroviral treatment. The regimens contained AZT, TDF or ABC in combination tablets with lamivudine or emtricitabine. All three regimens also contained efavirenz. The patients were matched for age, ethnicity, sex and pre-treatment CD4 count and viral loads. None had experienced any AIDS defining conditions.

We have shown that at all time points patients receiving ABC displayed a markedly different pattern of gene expression within subcutaneous adipose tissue, with a large percent of the overall variance accounted for by ABC compared to the other two treatment groups. The differences were particularly marked at 6 months after treatment initiation but were still evident at 18–24 months. After 6 months of treatment, 4185 genes showed significant differentially expressed sequences (DET) in patients receiving ABC relative to the controls while the number of DETs in the AZT and the TDF group were 895 and 381 respectively. After 18–24 months of treatment the differences between the three groups had narrowed. (Table 1)

We next examined the specific pathways over expressed in the three treatment groups compared to controls and to treatment naïve HIV-infected patients. All groups showed an enrichment of PPAR signalling and biosynthesis of fatty acids in agreement with previous studies of the effect of antiretroviral therapy [19,20,29,30] and a return to health.[8,28] The ABC group, however showed enrichment of a number of pathways at both time points that was not observed with the other two groups. The pathways of interest are involved with cell processes and cell communication (adherence junction, focal adhesions, tight junctions) and environmental information processing (WNT signalling, ECM receptor interaction, leukocyte transendothelial migration, neuroactive ligand interactions, MAPK signalling). Some of these may have important roles in cardiovascular disease.[31–35]

With the greatly increase survival observed with the use of combination anti-retroviral therapy long term complications of such therapies have assumed increasing importance. HIV itself [36] and antiviral treatments are associated with an increased incidence of cardiovascular disease [10] which is not entirely explained by traditional risk factors including adjustment for lipids [11]. In large cohort studies the increased risk of cardiovascular disease was associated with the use of protease inhibitors [10] and current or recent use of ABC and less consistently, didanosine.[10–14,18] Pooled analysis of two cohort studies [11,14] demonstrated a relative risk of 1.91 (95% CI: 1.5, 2.4) for use of ABC within previous six months and risk of myocardial infarction [37]. Other cohort studies [18,38] or randomised control trials (RCT) [16,39] have, on the other hand, not confirmed the association between ABC and cardiovascular events. Patients recruited in RCT, however, tend to be younger and followed for a shorter period of time than cohort studies. Hence despite their limitations results from large cohort studies cannot be entirely ignored [37]. Current International AIDS Society – USA Panel guidelines [40] advise avoidance of ABC use in patients at high risk of cardiovascular disease.

Possible mechanisms for the cardiovascular effects of ABC include an association with inflammatory markers,[41–43] abnormalities in endothelial cell function, [32,44] and platelet dysfunction. [45–48] These results have not been confirmed by other studies.[49–52] Thus the potential role of ABC in cardiovascular disease and the possible mechanisms through which this can be effected remain to be established.

The observation that the increased risk of cardiovascular events was only seen with recent or current use of ABC,[11,14,18,41] particularly in those in the higher Framingham risk group[11] suggests an additive effect of ABC to an already pre-existing underlying pathological mechanisms such as plaque instability, endothelial function, or thrombosis.[53] Our study provides another possible mechanism that requires further research. Tight junctions (TJ) focal adhesions and adherence junctions (AJ) were overrepresented in the KEGG pathway and ‘biological adhesions’ in GO terms 6 months after ABC treatment. In particular TJ and AJ are interlinked and important for maintaining tissue architecture [33,54,55] and have been implicated in cardiovascular disease. The gene VE-cadherin at AJ up-regulates TJ gene claudin-5 by limiting the nuclear accumulation of beta-catenin, which represses the claudin-5 promoter.[54] We showed down regulation of claudin-5 (CLDN5) and up-regulation of catenins (CTNNA1, CTNNB1) compared to controls and to AZT and TDF at 6 months (Tables 3 and 5) while there was no observable change in the expression of VE-cadherin (data not shown). These changes may result in increased endothelial permeability in subcutaneous adipose tissue in patients treated with ABC.[54] Were such time related changes in vascular permeability to be replicated in other tissues, and in particular coronary vasculature, an additional possible mechanism for the observed increase in the incidence of cardiovascular disease in patients starting abacavir treatment with underlying cardiovascular risk would be suggested. Further studies could provide insight into this suggested mechanism.

Pathways involved with environmental information processing (ECM-receptor interactions, MAPK signalling, neuroactive ligand interaction, Wnt signalling pathway) tended to be either enriched earlier in ABC (Table 2) or were significantly different at 6 months (Tables 4 and 5) and GO term ‘biological adhesions’ ‘cell adhesion’ and ‘cell matrix adhesion’, ‘cell-cell signaling’ and ‘signaling’ were overrepresented in the ABC group compared to the other two groups.

Cell adhesion molecules (CAMs) [31,33] and inflammatory markers are also believed to play an important role in atherosclerosis. However, reports of abnormalities in circulatory CAMs and inflammatory markers in endothelial function after ABC treatment are conflicting.[42,42,44,51,52,56–58] as are the nature of the relationship between circulating inflammatory markers and the expression of adhesion molecules at the endothelial surface.[34,59,60]

De Pablo et al. reported that ABC significantly induces leucocyte accumulation through activation of MAC-1 which reacts with its endothelial ligand ICAM-1 both *in vitro* and *in vivo*[56,61]. The demonstration of enrichment of genes involved with leukocyte transendothelial migration (Table 4) and up-regulation of ICAM 1, ICAM-4 and ICAM-5 compared to controls and, in the case of the latter two compared to the other two groups, after six-month ABC treatment (Table 5) are compatible with these findings.

Our study does have several limitations. Patients receiving AZT and TDF were randomised while those on ABC were only matched as to gender and ethnicity. While there was no statistical difference in weight (pre-treatment and at test), BMI, CD4 count, smoking history, and family history of diabetes and cardiovascular disease between the three groups, an unintended bias cannot be excluded. Moreover the number of subjects in each treatment group was necessarily small, and not all subcutaneous fat samples were available for study, further limiting the conclusions. However we adjusted for multiple comparisons by using the Benjamini-Hochberg False Discovery Rate (FDR) as well as Bonferroni multiple testing corrections. Finally as discussed above any extrapolation for gene expression from samples obtained from one tissue must only be guardedly extended to the whole subject, not least because we were unable to comment on post-transcriptional and regulation or post-translational modifications of gene expression.

Our study has highlighted novel mechanisms by which ABC may interfere with endothelial adhesion process and endothelial function in subcutaneous adipose tissue, particularly early in the treatment. In particular abnormalities in tight junction and adherence junction brought about by ABC use, if observed in other tissues, may be a contributing factor to cardiovascular events in persons already with some underlying risk for atherosclerosis[62], and may require further study.
